# PDE6δ-mediated sorting of INPP5E into the cilium is determined by cargo-carrier affinity

**DOI:** 10.1038/ncomms11366

**Published:** 2016-04-11

**Authors:** Eyad Kalawy Fansa, Stefanie Kristine Kösling, Eldar Zent, Alfred Wittinghofer, Shehab Ismail

**Affiliations:** 1 Max Planck Institute of Molecular Physiology, Otto-Hahn-Strasse 11, 44227 Dortmund, Germany; 2 CR-UK Beatson Institute, Garscube Estate Switchback Road, Glasgow G61 1BD, UK

## Abstract

The phosphodiesterase 6 delta subunit (PDE6δ) shuttles several farnesylated cargos between membranes. The cargo sorting mechanism between cilia and other compartments is not understood. Here we show using the inositol polyphosphate 5′-phosphatase E (INPP5E) and the GTP-binding protein (Rheb) that cargo sorting depends on the affinity towards PDE6δ and the specificity of cargo release. High-affinity cargo is exclusively released by the ciliary transport regulator Arl3, while low-affinity cargo is released by Arl3 and its non-ciliary homologue Arl2. Structures of PDE6δ/cargo complexes reveal the molecular basis of the sorting signal which depends on the residues at the −1 and −3 positions relative to farnesylated cysteine. Structure-guided mutation allows the generation of a low-affinity INPP5E mutant which loses exclusive ciliary localization. We postulate that the affinity to PDE6δ and the release by Arl2/3 in addition to a retention signal are the determinants for cargo sorting and enrichment at its destination.

## Abstract (web summary)

PDE6δ regulates the sorting of prenylated cargo proteins. Here Fansa *et al*. propose that the affinity of the interaction between PDE6δ and its cargo protein determines whether they are released by cytoplasmic or cilia-specific release factors ultimately determining their subcellular localization.

## Graphical abstract

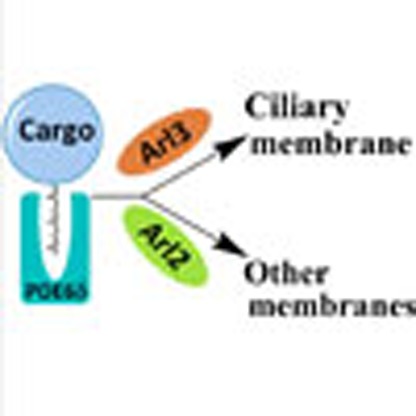

Primary cilia are antenna-like microtubule-based cell surface protrusions which can be found on eukaryotic cells and serve as sensory organelles. Genetic disorders affecting structure or function of cilia result in a large number of diseases collectively termed ciliopathies^1^,^2^. While the cilium appears as a protrusion in the plasma membrane that is open to the cell body, the ciliary content and membrane composition are different than that of the cell body and plasma membrane^3^,^4^. This is in part achieved by the presence of a diffusion and transport barrier, where entry and exit decisions of ciliary components have to be taken^5^,^6^.

PDE6δ is a prenyl-binding protein that was originally discovered as the delta subunit of rod photoreceptor-specific phosphodiesterase PDE6 (ref. 7). It was found as a solubilizing factor for the prenylated subunits of this enzyme and was later shown to be a general prenyl-binding protein (hence also called PrBP/PDE6δ)^8^,^9^,^10^,^11^. PDE6δ was shown to bind prenylated peptides or proteins of the Ras subfamily with approximately micromolar affinity^12^,^13^ and to play a critical role in their cellular distribution^14^,^15^,^16^. Since it is believed to be crucial for the localization and thus the activity of the oncoprotein Ras, inhibitors of the Ras-PDE6δ complex were actually considered as promising Ras drug candidates^17^.

INPP5E belongs to the inositol polyphosphate 5′-phosphatase family that hydrolyzes the 5′-phosphate of phosphatidylinositols and localizes to primary cilia^18^,^19^. The importance of the 5′-phosphatase activity for ciliary function is underscored by the finding that *INPP5E* is mutated in Joubert syndrome, a ciliopathy characterized by motor and intellectual disabilities^18^,^19^,^20^, and that the gene mutated in the OCRL (Oculocerebrorenal) or Lowe syndrome also encodes an inositol polyphosphate 5′-phosphatase^21^,^22^. INPP5E contains a C-terminal CaaX motif where the C-terminal residue Cys644 is farnesylated^23^. A mutation encoding a stop codon near to the CaaX motif (Q627) of INPP5E was identified in a family with MORM syndrome^18^, a ciliopathy characterized by intellectual disability, obesity, retinal dystrophy and micropenis^24^. This mutation was shown to affect INPP5E ciliary localization, which in combination with other reports^25^ indicates the importance of the C-terminus and its farnesylation for the ciliary localization of INPP5E (ref. 18).

Recently, PDE6δ was co-purified with INPP5E and siRNA-mediated knockdown of *PDE6δ* resulted in impaired ciliary localization of INPP5E (ref. 26). Moreover, a *PDE6δ* deletion mutation, which was identified in Joubert syndrome, was shown to impair the targeting of farnesylated INPP5E protein to the primary cilium^25^. Knockdown of *PDE6δ* also impeded the transport of GRK1 and PDE6 catalytic subunits to photoreceptor outer segments, which are considered specialized forms of cilia^27^,^28^.

The homologous small Arf-like GTP-binding proteins Arl2 and Arl3 have been shown to act as nucleotide-dependent-specific release factors of farnesylated cargo from PDE6δ *in vitro* and *in vivo*. Structural and kinetic analyses have shown that Arl2/3 act allosterically to increase the dissociation rate constants for cargo-carrier complexes^13^,^15^,^29^,^30^. In contrast, it was shown recently by pull-down experiments with cellular extracts that Arl3 but not Arl2 can efficiently release INPP5E from its complex with PDE6δ (ref. 25).

In analogy to nuclear localization signals a number of different ciliary localization signals have been identified for different transmembrane proteins^31^,^32^,^33^. However, not much is known about the molecular mechanism of how these signals are recognized and how decisions on ciliary entry based on these signals are made. For certain membrane-associated, post-translationally modified proteins carrying an N-terminal myristoyl or a C-terminal prenyl motif, it has been shown that the import into cilia is dependent on the carrier proteins PDE6δ, UNC119a and UNC119b and on Arl3 as displacement factor^13^,^25^,^28^,^30^,^34^. However, it has been extensively documented that Ras proteins as well as Rheb require PDE6δ for their proper localization at the plasma membrane or internal membranes, but do not appear to be localized in cilia^15^,^16^.

This begs the question about the mechanism of PDE6δ-mediated sorting of farnesylated cargo between the cilium and other cellular compartments. Thus, we set out to investigate the molecular basis of farnesylated cargo sorting using ciliary INPP5E and non-ciliary Rheb as an example. Here, we show that a 100-fold difference in the binding affinity of farnesylated cargo with PDE6δ and the specific release of high-affinity cargo by activated Arl3**·**GTP determines cargo sorting into cilia, while low-affinity cargo can be released by both Arl3**·**GTP and Arl2**·**GTP and stays outside the cilium. Moreover, we show by structural, biochemical and cell biological approaches, how and why the binding affinity is dependent on the residues at the −1 and −3 positions preceding the farnesylated cysteine and that sorting of farnesylated cargo can be manipulated by changing the affinity to PDE6δ.

## Results

### INPP5E and Rheb localization and binding affinity to PDE6δ

**Figure 1:**
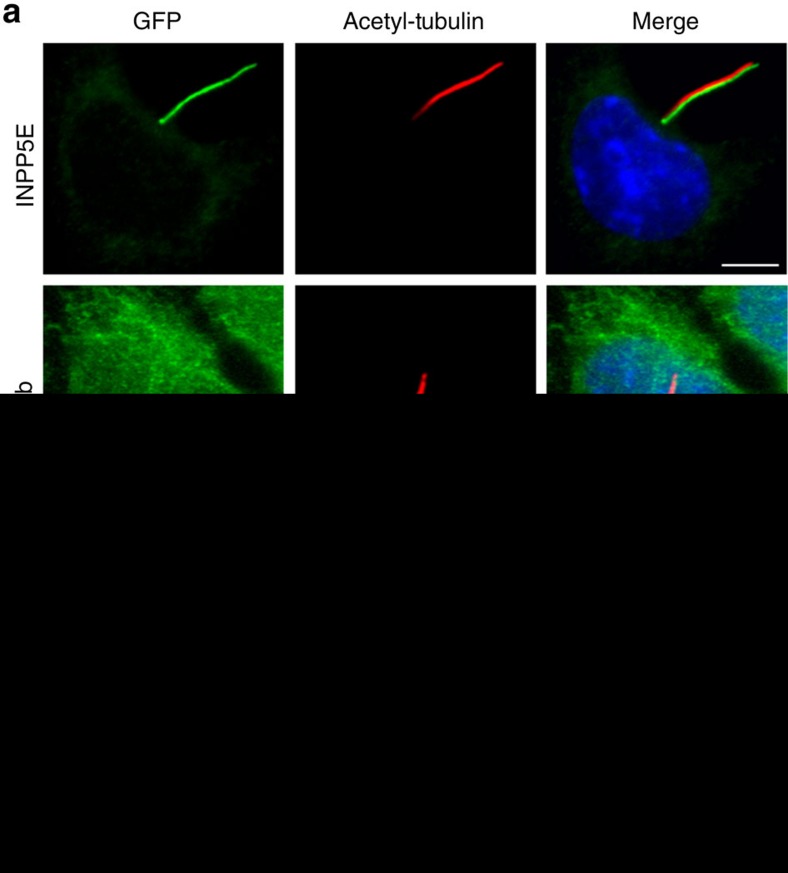
Localization of INPP5E and Rheb and their affinity to PDE6δ. (**a**) Localization of INPP5E and Rheb in IMCD3 cells. Stably expressed GFP-INPP5E colocalizes with acetylated tubulin, as shown by immunostaining of acetylated tubulin (red) and GFP fluorescence (LAP-tagged) (green), while GFP-Rheb (green) localizes to endomembranes and is almost absent from cilia. White bar indicates 5 μm. (**b**) 0.01 μM TAMRA-labelled farnesylated peptide (SQNSSTIC(Far)-OMe) from INPP5E (left) and 0.5 μM FITC-labelled peptide (SQGKSSC(Far)-OMe) from Rheb (right) were titrated with increasing concentrations of PDE6δ and the increase in fluorescence polarization was plotted against the PDE6δ concentration. The data were fitted to a quadratic equation giving the indicated dissociation constants (*K*_d_).±indicates s.d. (*n*≥9).

Using IMCD3 cells stably expressing either *INPP5E* or *Rheb* fused to a localization and tandem affinity purification (LAP) tag^35^, we can show that INPP5E localizes almost exclusively to the primary cilium with very small fraction in the cell body (Fig. 1a; upper), which is consistent with previous reports^18^,^19^,^26^. In contrast, Rheb mainly localizes to endomembranes (Fig. 1a; lower), this observation is consistent with previous reports^13^,^36^. Given that the prenyl-binding protein PDE6δ is the shuttle factor mediating the localization of INPP5E and Rheb^13^,^16^,^18^,^25^,^26^, we set out to characterize the interaction of PDE6δ with INPP5E and Rheb. Previously we have shown that farnesylated C-terminal peptides derived from Rheb or KRas bind to PDE6δ in exactly the same way and with similar affinities as the full-length farnesylated proteins^12^,^13^. Hence, we used a fluorescently labelled C-terminal farnesylated and carboxy-methylated peptide of INPP5E (residues 637–644) and Rheb (residues 175–181) to measure the affinity to PDE6δ by fluorescence polarization. Figure 1b (left) shows that PDE6δ binds to INPP5E peptide with low nanomolar affinity (*K*_d_=3.7 nM±0.2,±indicates s.d., *n*=9). In contrast, the affinity between PDE6δ and the farnesylated C-terminal peptide of Rheb falls into the submicromolar range (*K*_d_=445±83 nM,±indicates s.d., *n*=10) (Fig. 1b; right), which is in the same range with the previously described values^12^,^13^. These data raised the question, whether the almost 100-fold higher affinity of INPP5E towards PDE6δ as compared to Rheb is involved in the sorting mechanism of these two proteins to different destinations.

### High-affinity cargo is specifically released by Arl3·GTP

**Figure 2:**
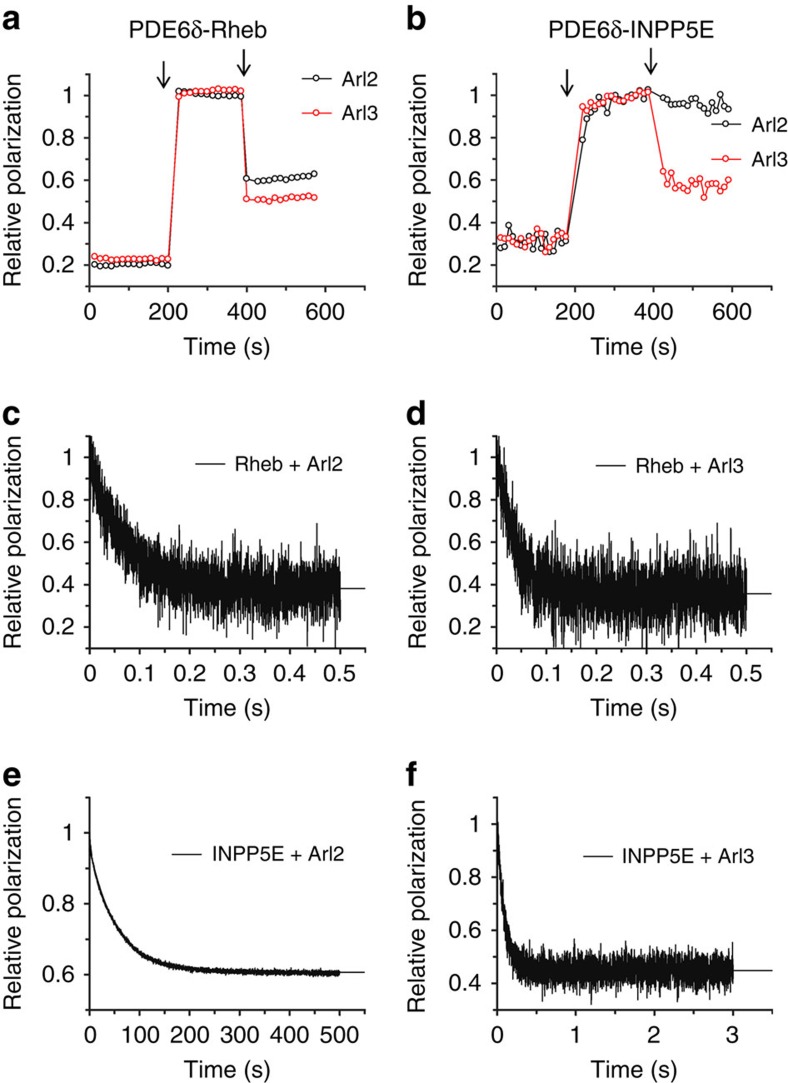
INPP5E release from PDE6δ by Arl2·GppNHp and Arl3·GppNHp. (**a**) Fluorescence polarization measurements of 0.5 μM FITC-labelled Rheb peptide followed by addition of 0.5 μM PDE6δ and the addition of 5 μM Arl2·GppNHp or Arl3·GppNHp (arrow). (**b**) Fluorescence polarization measurements of 0.2 μM TAMRA-labelled INPP5E peptide followed by the addition of 0.2 μM PDE6δ (arrow) and 5 μM Arl2·GppNHp or Arl3·GppNHp (arrow). (**c**–**f**) Stopped-flow fluorescence polarization kinetic experiment where complexes of 1 μM PDE6δ with either 0.2 μM of FITC-labelled Rheb peptide (**c**,**d**) or TAMRA-labelled farnesylated INPP5E peptide (**e**,**f**) were mixed with 100-fold excess of unlabelled peptide and 10 μM of Arl2 (**c**,**e**) or Arl3 (**d**,**f**) as indicated.

Towards an explanation for the possible sorting mechanism that leaves some PDE6δ-cargo in the cell body but allows others to be enriched in the cilia we turned to the release activities of Arl2 and Arl3. Both GTP-binding proteins in their active conformation have been shown to be responsible for releasing cargo from PDE6δ. While Arl2 is a non-ciliary protein, Arl3 localizes along the length of the cilium^37^. Using fluorescence polarization, we measured the release of INPP5E and Rheb peptides from PDE6δ by the addition of Arl2 or Arl3 bound to the non-hydrolysable GTP analogue GppNHp. The data show that Rheb peptide can be released by both Arl2**·**GppNHp and Arl3**·**GppNHp (Fig. 2a), supporting earlier observations^13^. In contrast, INPP5E peptide can only be released by Arl3**·**GppNHp under the same conditions (Fig. 2b). To compare the cargo release kinetics of Arl2**·**GppNHp and Arl3**·**GppNHp, we measured the dissociation rate constants of INPP5E and Rheb peptides from PDE6δ in the presence and absence of Arl3**·**GppNHp or Arl2**·**GppNHp, by adding a large excess of unlabelled peptide to silence the back reaction. In the absence of Arl2/3, Rheb showed an intrinsic dissociation rate (*k*_off_=0.95±0.004 s^−1^,±indicates s.d., *n*=4), while no measurable dissociation rate could be observed for INPP5E in a reasonable time window. This observation is in line with the almost 100-fold difference in the binding affinity between both peptides determined from the steady state equilibrium measurements. The presence of Arl3**·**GppNHp or Arl2**·**GppNHp has a similar acceleration effect on the dissociation rate of Rheb peptide from PDE6δ (*k*_off_=27.2±0.7 and 15.3±0.3 s^−1^, respectively, ±indicates s.d., *n*=4) (Fig. 2c,d). However, the release of INPP5E peptide in the presence of Arl3**·**GppNHp shows an estimated 10,000-fold acceleration (*k*_off_=10.7±0.2 s^−1^,±indicates s.d., *n*=4), while release by Arl2**·**GppNHp (*k*_off_=0.018±0.0005, s^−1^,±indicates s.d., *n*=4) is almost 600-fold slower (Fig. 2e,f). Taken together, our data suggest that high-affinity farnesylated cargo can be specifically released by Arl3, while low-affinity cargo can be released similarly by both Arl2 and Arl3.

### Role of Arl3 N-terminal helix in the release mechanism

**Figure 3:**
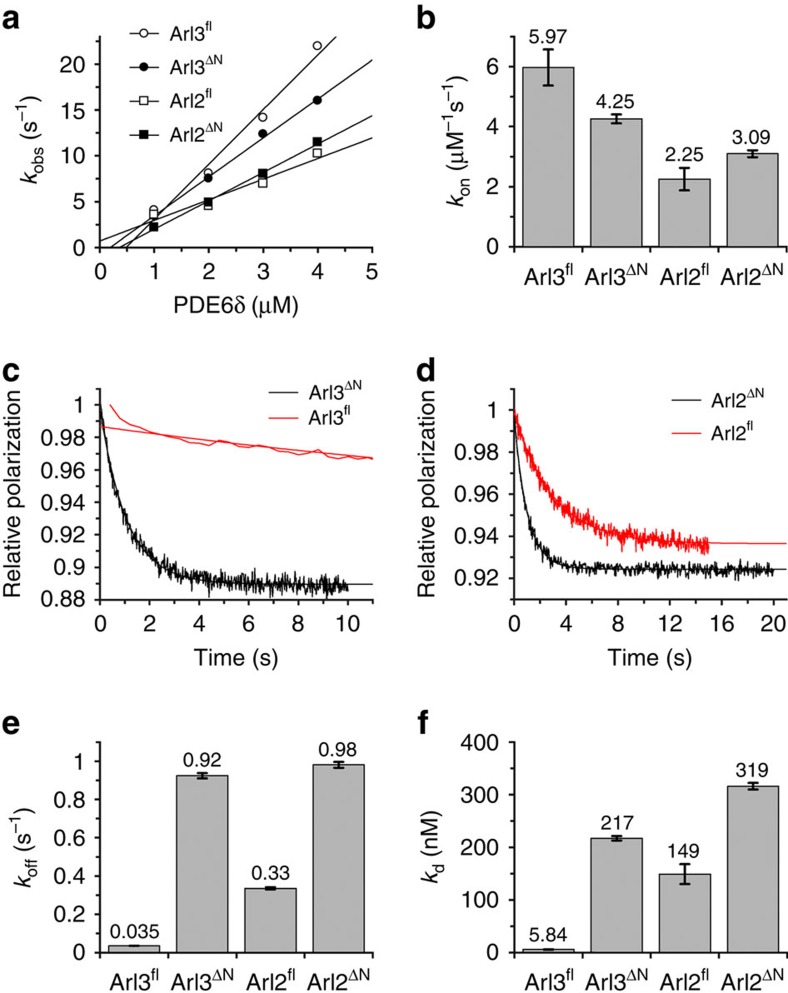
Contribution of the N-terminal helix of Arl3 to the binding affinity with PDE6δ. (**a**) Stopped-flow fluorescence polarization kinetic measurements of the association of 0.2 μM mantGppNHp loaded Arl proteins with increasing concentrations of PDE6δ. The observed pseudo-first order rate constants (*k*_obs_) are plotted against PDE6δ concentration. (**b**) Bar charts of the association rate constants (*k*_on_) determined in **a**. (**c**,**d**) Stopped-flow fluorescence polarization kinetic experiments where complexes of 2 μM PDE6δ with 0.2 μM of full-length (Arl^fl^) or N-terminally deleted (Arl^ΔN^) mantGppNHp loaded Arl proteins as indicated were mixed with 200-fold excess of unlabelled Arl proteins to determine *k*_off_. (**e**) Bar charts of the dissociation rate constants (*k*_off_) from experiments in **c**,**d**. (**f**) Bar charts of the equilibrium dissociation constants (*K*_d_) of complexes between PDE6δ and Arl proteins as determined from the kinetic constants in **c**,**e**. Error bars indicate s.d., *n*=4.

Previously we have shown that the N-terminal helix of Arl3 is important to release myristoylated cargo from a complex with the shuttle factor UNC119 (ref. 30). To find out whether the N-terminus of Arl3 and/or Arl2 has a similar if any role in the interaction with PDE6δ, fluorescence polarization measurements using full-length Arl3 (Arl3^fl^) or an N-terminal truncated form (Arl3^ΔN^) were performed. Supplementary Fig. 1 shows that Arl3^ΔN^ is unable to release the INPP5E peptide from PDE6δ as compared with Arl3^fl^. To investigate the role of the N-terminal helix of Arl3 in the release mechanism, we measured association and dissociation rate constants to determine the affinity of PDE6δ towards Arl2 and Arl3 in both full-length and N-terminal truncated forms. Association rate constants between the four proteins Arl3^fl^, Arl3^ΔN^, Arl2^fl^ and Arl2^ΔN^ are rather similar although association is almost twice as fast for full-length Arl3 as compared with Arl2 (Fig. 3a,b). In contrast, determination of the dissociation rate constants shows large differences. While the difference in *k*_off_ between full-length protein Arl2^fl^ and N-terminal deleted Arl2^ΔN^ is only threefold, Arl3^fl^ shows a 26-fold higher residence time with PDE6δ, as compared with Arl3^ΔN^ (Fig. 3c–e). By calculating the equilibrium dissociation constants (*K*_d_=*k*_off_/*k*_on_), Arl3^ΔN^, Arl2^fl^ and Arl2^ΔN^ exhibit affinities in the submicromolar range (217±4.3, 149±19 and 316±6.3 nM, respectively,±indicates s.d., *n*=4), whereas Arl3^fl^ has an affinity in the low nanomolar range (*K*_d_= 5.8±0.5 nM,±indicates s.d., *n*=4) (Fig. 3f). The *K*_d_ values for Arl2^fl^ and Arl3^fl^ differ from previously determined values^38^, likely because of the different techniques used.

Our data suggest that the N-terminal helix of Arl3 makes a significant contribution to the interaction with PDE6δ and increases the affinity between the proteins by 37-fold. This additional input of Arl3 compared with Arl2 is probably a major factor in the ability of Arl3 to release high-affinity farnesylated cargo from PDE6δ. A similar effect was shown for the Arl3/UNC119 complex where in contrast to Arl2 (and any other Arf protein), the N-terminal helix of Arl3 did not detach from the surface of the protein after the GDP-GTP conformational change and actively participates in the release mechanism in the closed position^30^.

### The sorting signal of PDE6δ-related farnesylated cargo

**Figure 4:**
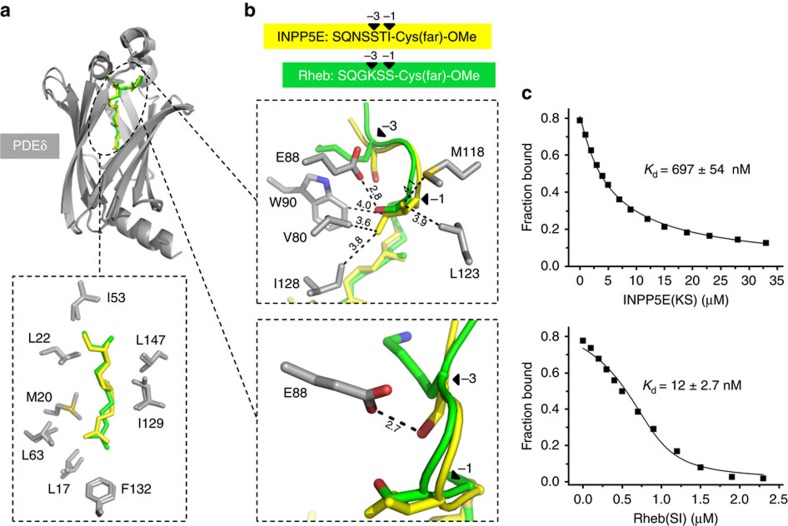
Structural analysis of the interaction between PDE6δ and farnesylated cargo. (**a**) Superimposition of farnesylated INPP5E peptide-PDE6δ and Rheb-PDE6δ (PDB code: 3T5G). The farnesyl moieties of INPP5E (yellow) and Rheb (green) insert into the hydrophobic pocket of PDE6δ (grey) (upper) and maintain the same interaction pattern with the surrounding hydrophobic residues of PDE6δ (lower). (**b**) Differences in the interaction pattern at the −1 (upper) and −3 (lower) residues from Rheb (green) and INPP5E (yellow) with PDE6δ, bond lengths (black dashed line) are given in Å. (**c**) Titrations of a complex between 0.5 μM FITC-labelled Rheb peptide and 1 μM PDE6δ with increasing concentrations of INPP5E(KS) (upper) and Rheb(SI) (lower) mutant peptides. Titration data were fitted with a competition model. ±indicates s.d. (*n*≥12).

To investigate the nature of the affinity difference between INPP5E and Rheb peptides towards PDE6δ in more details, we solved the crystal structure of the INPP5E peptide in complex with PDE6δ at 1.85 Å resolution (data collection and refinement statistics summarized in Supplementary Table 1). Superimposition of the INPP5E peptide/PDE6δ complex with the structure of PDE6δ in complex with Rheb (PDB code: 3T5G) shows that the immunoglobulin-like β-sandwich folds of PDE6δ overlay well with an r.m.s. deviation of 0.5731 Å. The proteins show a hydrophobic cavity, where the farnesyl moieties of INPP5E and Rheb are inserted (Fig. 4a; upper). The prenyl groups overlay well and make an identical interaction pattern with the surrounding hydrophobic residues of PDE6δ (Fig. 4a; lower). However, the side chains of the residues on the −1 and −3 positions upstream of the farnesylated cysteine (the 0 position) in INPP5E and Rheb show different contacts with PDE6δ. As shown in Fig. 4b (upper), the serine side chain of Rheb on the −1 position makes a hydrogen bond with the side chain of glutamic acid (Glu88) from PDE6δ, whereas the hydrophobic side chain of the isoleucine of INPP5E at the equivalent position is situated in a highly hydrophobic environment mediated by five hydrophobic residues of PDE6δ (Val80, Trp90, Met118, Leu123 and Ile128). On the other hand, the lysine side chain of Rheb at the −3 position is pointing away from the binding pocket of PDE6δ, while the serine side chain of INPP5E at the equivalent position makes a hydrogen bond with the side chain of glutamic acid (Glu88) (Fig. 4b; lower).

Thus, we reasoned that the different contact patterns of INPP5E and Rheb peptides with PDE6δ are responsible for the difference in affinities. To prove this, we generated two peptides, where the amino acids on the −1 and −3 positions were swapped between INPP5E and Rheb, creating INPP5E(KS) (S641K/I643S) and Rheb(SI) (K178S/S180I) peptides. Affinities of the swapped peptides to PDE6δ were determined by titrating increasing amounts of unlabelled INPP5E(KS) and Rheb(SI) into a preformed complex of fluorescent Rheb peptide with PDE6δ and monitoring the displacement by the decrease in fluorescence polarization. Analysis of the data with a competition model derived from the law of mass action as described^17^,^39^ shows that the affinities to PDE6δ can be reversed, with a *K*_d_ values of (697±54 nM,±indicates s.d., *n*=14) for INPP5E(KS) and (12±2.7 nM,±indicates s.d., *n*=12) for Rheb(SI) (Fig. 4c).

To confirm the conclusion relating to the −1 and −3 positions, we measured the affinities of farnesylated peptides derived from rhodopsin kinase GRK1 and the γ-subunit of transducin GNGT1 (Tγ) with PDE6δ. It is important to note that, GRK1 carries Met and Ser at −1 and −3 positions similarly with INPP5E, whereas GNGT1 (Tγ) carries Gly and Lys at −1 and −3 positions similarly with Rheb (Supplementary Fig. 2a). The results showed high binding affinity (7.2±1.3 nM,±indicates s.d., *n*=12) of GRK1 and low binding affinity (6,573±477 nM,±indicates s.d., *n*=9) of Tγ for PDE6δ (Supplementary Fig. 2b). These data suggest that the binding affinity between PDE6δ and farnesylated cargo is dependent on the sequence of the farnesylated C-terminus, in particular on the −1 and −3 positions relative to the farnesylated cysteine.

### Dependency of INPP5E ciliary localization on PDE6δ and Arl3

**Figure 5:**
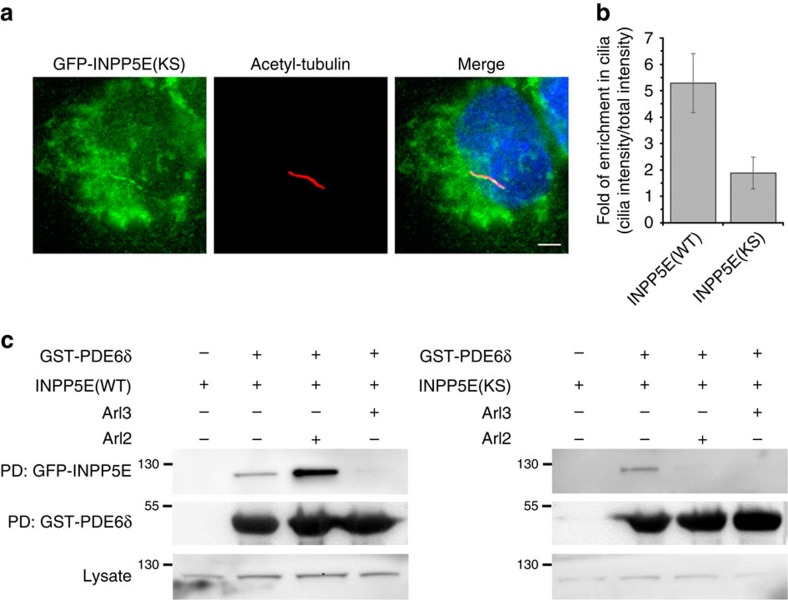
INPP5E ciliary enrichment is dependent on high affinity to PDE6δ. (**a**) Localization of INPP5E(KS) mutant (green) in IMCD3 cells which were stably transfected with the GFP-tagged protein. White bar indicates 5 μm. (**b**) Bar chart showing ratio of GFP intensity in cilia to the total GFP intensity, indicating the enrichment of GFP-tagged protein in cilia. Data have been collected for 43 cells of INPP5E(WT) and 35 cells for INPP5E(KS) and analysis was performed using CellProfiler. Error bars indicate s.d., *n*≥35 (*P*<0.05; Student’s *t*-test). (**c**) GST pull-downs were performed using GST-PDE6δ along with the IMCD3 cell lysates stably expressing INPP5E(WT) (left) or INPP5E(KS) (right). Formed complexes in the pulldown experiment were incubated with Arl2 or Arl3 as indicated. The amount of GFP-tagged interacting proteins bound to GST-PDE6δ was detected by immunoblotting with an antibody against GFP.

To test whether reducing the affinity of INPP5E to PDE6δ is affecting its ciliary localization, we stably transfected the INPP5E(KS) mutant into IMCD3 cells and compared its localization with INPP5E(WT). Figure 5a shows that INPP5E(KS) mutant is not enriched in cilia anymore but is localized all over the cell including the cilium, while INPP5E(WT) is highly enriched in cilia with only a minor fraction in the cell body (Fig. 1a). Evaluation of mean fluorescence intensity ratio between cilia and whole cell shows that INPP5E(WT) has a 5.3-fold enrichment in the cilia, while the INPP5E(KS) mutant loses its ciliary enrichment and is more evenly distributed over the entire cell (Fig. 5b and Supplementary Fig. 3).

**Figure 6:**
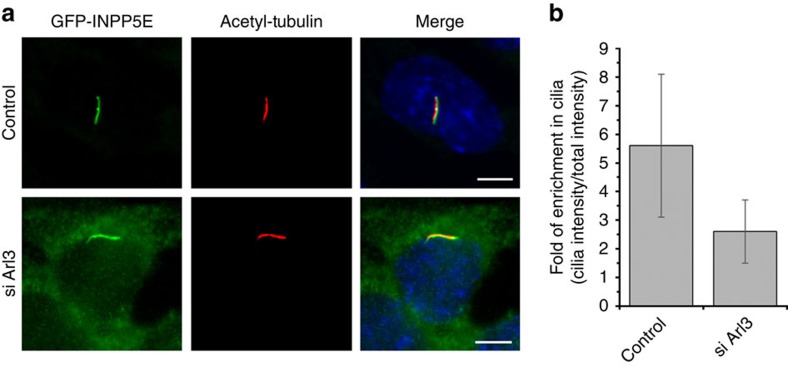
INPP5E ciliary enrichment is dependent on Arl3. (**a**) Localization of INPP5E (green) in IMCD3 cells which were stably transfected with the LAP-tagged protein followed by the transfection with either negative control siRNA or siRNA directed against *Arl3*. White bar indicates 5 μm. (**b**) Bar chart showing ratio of GFP intensity in cilia to the total GFP intensity, indicating the enrichment of GFP-tagged protein in cilia. Data have been collected for 90 cells which were treated with control siRNA and for 82 cells which were treated with siRNA against *Arl3* and analysis was performed using CellProfiler. Error bars indicate s.d., *n*≥82 (*P*<0.05; Student’s *t*-test).

We propose that the mislocalization of INPP5E(KS) mutant could result from its weak affinity to PDE6δ, which enables its release by Arl2 outside the cilium, resulting in its retention at the endomembranes. To support this assumption, we used the stably transfected IMCD3 cells expressing INPP5E(WT) or mutant INPP5E(KS) and performed a GST pull-down experiment with PDE6δ in the presence and absence of Arl3**·**GppNHp or Arl2**·**GppNHp. The results show that the INPP5E(KS) mutant can indeed be released by both Arl2**·**GppNHp and Arl3**·**GppNHp, while INPP5E(WT) is specifically released only by Arl3**·**GppNHp (Fig. 5c). Confirming with this, siRNA-mediated knockdown of *Arl3* shows loss of dominant ciliary localization of INPP5E and its redistribution between cilia and cellular endomembranes (Fig. 6 and Supplementary Fig. 4).

**Figure 7:**
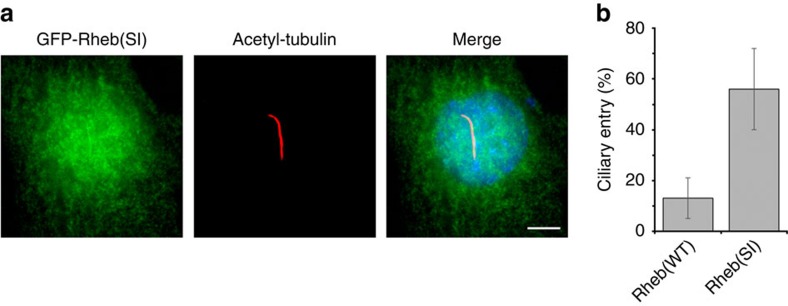
Ciliary entry of Rheb is dependent on the affinity to PDE6δ. (**a**) Localization of Rheb(SI) mutant (green) in IMCD3 cells which were stably transfected with the GFP-tagged protein. White bar indicates 5 μm. (**b**) Bar chart showing the percentage of Rheb-positive cilia. Data were collected from two independent experiments for each Rheb(WT) (38 and 87 cells per experiment) and Rheb(SI) (92 and 126 cells per experiment). Error bars indicate s.d., *n*≥125 (*P*<0.05; Student’s *t*-test).

In line with these experiments, we tested whether increasing the affinity of Rheb to PDE6δ permits its ciliary entry. For this we stably transfected the Rheb(SI) mutant into IMCD3 cells and compared its localization to that of Rheb(WT). Rheb(SI) showed a more than fourfold increase in ciliary localization as compared with Rheb(WT) (Fig. 7). This result indicates that increasing the affinity of Rheb towards PDE6δ shifts the equilibrium of Rheb distribution towards the cilium as compared to the entire cell. The non-exclusive ciliary localization of Rheb(SI) mutant could be explained by the absence of a Rheb specific retention signal inside the cilia.

Taken together, our data suggest that the high binding affinity between INPP5E and PDE6δ and the specific release by Arl3**·**GTP are essential determinants for the ciliary localization of INPP5E.

## Discussion

Consistent with our previous reports^12^,^13^, here we show that non-ciliary farnesylated cargo such as Rheb binds to PDE6δ with submicromolar affinity. Interestingly, the binding affinity between PDE6δ and the ciliary farnesylated protein INPP5E is in the low nanomolar range. Structural analysis revealed that the residues at the −1 and −3 positions relative to the farnesylated cysteine are the determinants for the binding affinity to PDE6δ. This finding was confirmed by mutational analysis and by the binding affinity measurements of farnesylated peptides derived from rhodopsin kinase (GRK1) and the γ-subunit of transducin (Tγ). The high binding affinity of GRK1 to PDE6δ could explain its mislocalization in the outer segment of photoreceptor in the absence of PDE6δ, while Tγ, which has a low-affinity to PDE6δ, is only minimally affected^28^. The latter suggests that another farnesyl binding protein might exist to take over the role as a shuttle factor for Tγ or that the ciliary entry of the heterotrimeric transducin does not rely solely on the farnesylated γ-subunit. Our findings suggest that the affinity of farnesylated cargo is an essential determinant of its PDE6δ-mediated sorting into the ciliary compartment.

It has been reported that Arl3 is localized in the cytoplasm and inside cilia^37^, while no ciliary localization for Arl2 has been reported so far. Considering that the complex between high-affinity cargo such as INPP5E or GRK1 with PDE6δ can be released specifically by Arl3 and that both proteins are highly enriched in cilia, one would have to predict that the active GTP-bound form of Arl3 is only localized inside the cilium and thus is able to release cargo exclusively in this compartment. This assumption is supported by our recent study which showed that the ciliary protein Arl13B is the specific guanine nucleotide exchange factor for Arl3 (ref. 40) as well as by studies showing that retinitis pigmentosa 2 (RP2), the GTPase activating protein of Arl3, localizes at the basal body of the cilium or the preciliary region^41^,^42^, so that Arl3·GTP should reside exclusively inside the cilium and would get hydrolyzed to Arl3·GDP while exiting the cilium. Confirming with this, Arl3 does not seem to take over the role of Arl2 in releasing low-affinity farnesylated cytosolic cargo, as siRNA-mediated knockdown of Arl2 was shown to be sufficient to mislocalize KRas (ref. 15). Thus, our data suggest that high-affinity farnesylated cargo is specifically released by Arl3 inside cilia and Arl2 is specific for the release of low-affinity cargo outside cilia.

Our results are apparently not in agreement with previous results^25^,^26^, who showed that the transport of INPP5E is independent of Arl3. In these reports, data were analysed in terms of ciliary localization (INPP5E-positive cilia), not taking the distribution of INPP5E between cilia and the entire cell into account. Such analysis has enabled us to determine the fold enrichment of INPP5E inside cilia and how it is affected by either changing the affinity to PDE6δ or by Arl3 knockdown. The redistribution of INPP5E in the cells, which were treated with siRNA against *Arl3*, showed similar but generally weaker effect as compared with the redistribution of the low-affinity mutant INPP5E(KS) (Figs 5b and 6b). The effect of Arl3 knockdown might be limited by the incomplete knockdown and by the fact that staining of INPP5E inside cilia does not differentiate between free or PDE6δ-bound phosphatase.

Both ciliary cargo and Arl3 seem to bind to PDE6δ with high affinities, non-ciliary cargo and Arl2 on the other hand bind to PDE6δ with low affinities. Thus we assume that the cargo release by Arl3 inside cilia or Arl2 in the cytosol might not be complete at comparable concentrations of all components. As a consequence an additional signal would be required to drive the equilibrium to completion and to retain cargo at its destination. A retention signal could be achieved by the interaction with membrane or other interacting partners. The endomembrane system offers a large surface area and could play the role as retention signal for cytosolic farnesylated cargo such as Rheb. A possible ciliary retention signal for INPP5E could be Arl13B. The specific ciliary protein Arl13B has been shown to directly interact with INPP5E and its knockdown results in INPP5E mislocalization^26^.

**Figure 8:**
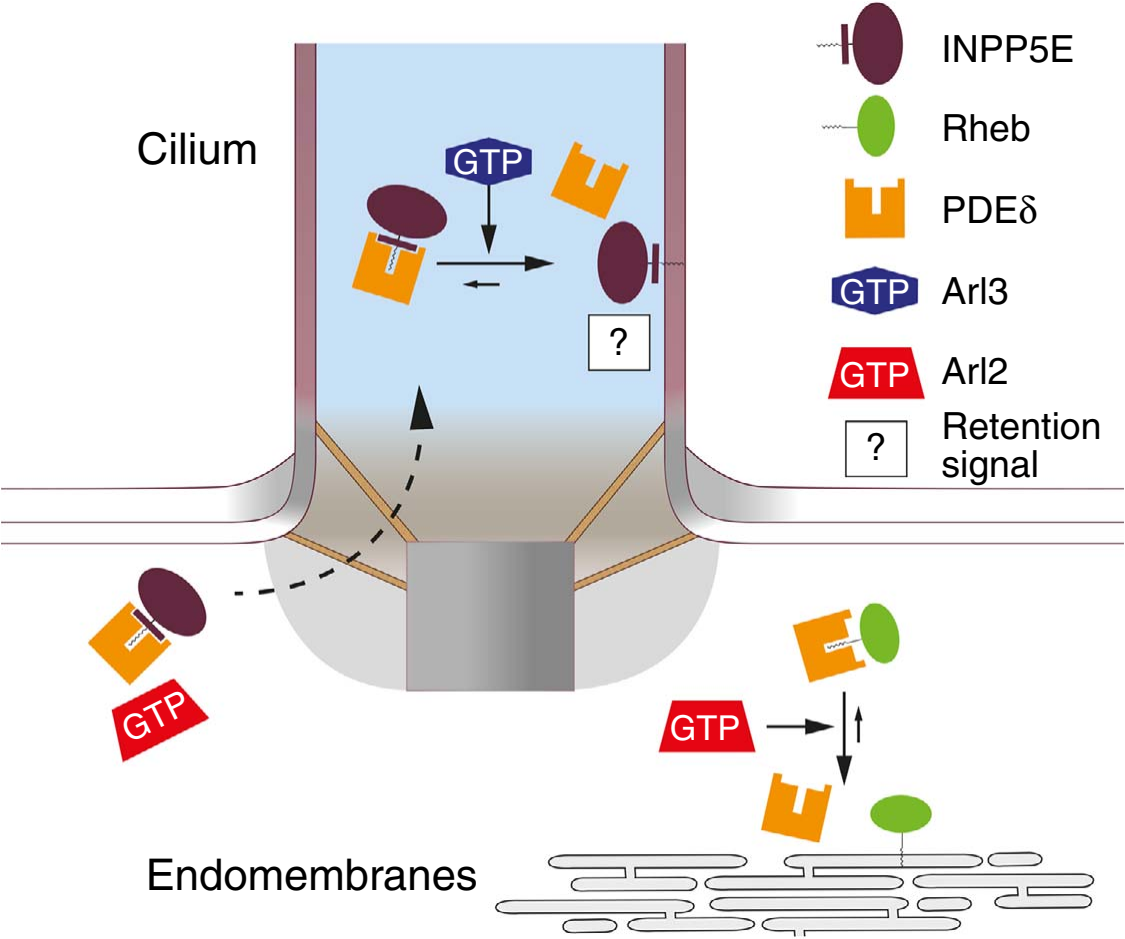
Model of PDE6δ-mediated sorting of farnesylated cargo. High-affinity cargo such as INPP5E can be specifically released from PDE6δ by Arl3·GTP in the cilium, but not by Arl2·GTP in the cytosol. In contrast, low-affinity cargo such as Rheb can be released by Arl2·GTP. As a consequence, PDE6δ-free INPP5E can be specifically retained and thus be enriched in the ciliary compartment while PDE6δ-free Rheb is retained at endomembranes and stays outside the cilia.

In this report, we propose a three step model for PDE6δ-mediated sorting of farnesylated cargo into different cellular compartments. The binding affinity of farnesylated cargo to PDE6δ is the first fundamental step in the sorting mechanism, followed by the specific release of high-affinity cargo by Arl3 inside cilia or the release of low-affinity cargo by Arl2 in the entire cell. Finally, a retention signal keeps the farnesylated cargo at its destination (Fig. 8). Interfering with any of these steps can provide valuable insights in studying the role of INPP5E in ciliopathies especially that a mutation which influences its localization to cilia is associated with MORM syndrome. Furthermore INPP5E localization studies for Arl13B patient mutations associated with Joubert syndrome will deepen our understanding of the molecular basis of ciliopathies. Finally it would be interesting to exploit available small molecules that inhibit the interaction of PDE6δ with farnesylated cargo in studying the role of INPP5E in cilia and ciliopathies.

## Methods

### Plasmids

Vectors for transfection of IMCD3 Flp-In cells were generated using the Gateway cloning technology (Life technologies) following the manufactureŕ;s recommendations. Mouse *INPP5E* and *Rheb* PCR fragments were amplified using the following primers: *INPP5E* (F-5′- ATGCCATCCAAGTCAGCTTGCCTG-3′, R-5′- TCAGGACACGGTGCAAACTGCACTGG-3′), *Rheb* (F-5′-ATGCCGCAGTCCAAGTCCCGGAAG-3′, R-5′- TCACATCACCGAGCATGAAGACTTGCC-3′). Entry clones were obtained by integration of the PCR fragments into pCR8/GW/TOPO vector (Life technologies). Mouse *INPP5E* and *Rheb* entry clones were located to pG-LAP3 destination vector (Addgene)^43^ by LR recombination. The pG-LAP3 vector encoded a LAP-tag (GFP-TEV-site-S-peptide) N-terminal to *INPP5E* and *Rheb*. *INPP5E S641K/V643S* (*INPP5E(KS)*) and *Rheb K178S/S180I (Rheb (SI))* clones were created using INPP5E-pG-LAP3 and Rheb-pG-LAP3 as template and following single mutagenesis primers: *INPP5E V643S* (F-5′-GCCAGAGCTCCAGTGCAAGTTGCACCGTGTCCTGAAAGGGCG-3′), *INPP5E S641K* (F-5′-GCCAGAGCTCCAAAGCAGTTTGCACCGTGTCCTGAAAGGGCG-3′). *Rheb K178S* (F-5′-GGGGCAGCTTCACAAGGCTCGTCTTCATGCTCGGTGATG-3′), *Rheb S180V* (F-5′-GCTTCACAAGGCTCGTCTGTATGCTCGGTGATGTGAAAGG-3′).

### Proteins

All proteins were expressed in *Escherichia coli* strain BL21-CodonPlus(DE3)-RIL. Cells were induced at OD ∼0.6 with 100 μM IPTG and incubated at 20 °C overnight. Cells were harvested and lysed in lyses buffer (25 mM Tris-HCl, pH 7.5, 150 mM NaCl and 1 mM b-mercaptoethanol, 1 mM PMSF) using French press. Supernatants of C-terminal histidine-tagged full-length Arl3, Arl2 and N-terminal histidine-tagged PDE6δ were loaded onto a Ni-NTA column (QIAGEN). Proteins were eluted with elution buffer (25 mM Tris-HCl, pH 7.5, 150 mM NaCl and 1 mM b-mercaptoethanol, 250 mM imidazole), followed by gel filtration on a Superdex 75 S26/60 column using elution buffer without imidazole. Supernatants of N-terminal GST-tagged truncated Arl3 and Arl2 (aa 18-177, 17-178, respectively) were expressed, harvested and lysed similar to the histidine-tagged proteins. The supernatants were loaded onto GSH-column (Amersham Biosciences). Proteins were eluted with elution buffer (25 mM Tris-HCl, pH 7.5, 150 mM NaCl and 1 mM b-mercaptoethanol, 20 mM glutathione). The GST-fusion proteins were separated from the tag by proteolytic cleavage followed by gel filtration on a Superdex 75 S26/60 column using elution buffer without glutathione. Nucleotide exchange of the GDP bound Arl3 and Arl2 proteins was achieved by overnight incubation at 4 °C with 4 U mg^−1^ alkaline phosphatase (Roche Diagnostics) and 1.5-fold excess of the non-hydrlysable GTP analogue (GppNHp) or the fluorescently labelled GppNHp (mantGppNHp) and followed by gel filtration.

### Peptides

Fluorescently labelled, farnesylated and carboxy-methylated Rheb peptide (Fluorescein-SQGKSSC(Far)-OMe) and INPP5E peptide (SQNSSTIC(Far)-OMe) were obtained from JPT. Farnesylated and carboxy-methylated Rheb(SI) (SQGSSIC(Far)-OMe), INPP5E(KS) (SQNSKTSC(Far)-OMe), GRK1 (SSSKSGMC(Far)-OMe) and Tγ (FKELKGGC(Far)-OMe) peptides were obtained from CambridgePeptides.

### Crystallization and structure determination

The INPP5E-peptide (SQNSSTIC(Far)-OMe) was dissolved in DMSO and mixed with 500 μM solution of PDE6δ at 1:1 molar ratio in a buffer containing 25 mM Tris-HCl (pH 7.5), 150 mM NaCl and 3 mM DTE. The crystals appeared in Protein Complex suite from Qiagen, 1.4 M sodium malonate (at 20 °C) and were flash frozen in a cryoprotectant solution that contains the mother liquor in addition to 16% (v/v) glycerol. Diffraction data set was collected at the X10SA beamline of the Suisse Light Source, Villigen. XDS program was used for data processing. The structure was solved by molecular replacement using Molrep from CCP4 (suite) and PDE6δ from the PDE6δ-farnesylated Rheb complex (PDB code: 3T5G) as a search model. The farnesylated INPP5E peptide was built using WinCoot and refinement was done with REFMAC5. Refinement and data collection statistics are summarized in Supplementary Table 1. Structure coordinates were deposited in the Protein Data Bank (PDB code 5F2U). A stereo image of a portion of the electron density map is displayed in Supplementary Fig. 5.

### Fluorescence polarization measurements

All fluorescence polarization measurements were performed at 20 °C in a buffer containing 25 mM Tris-HCl (pH 7.5), 50 mM NaCl and 3 mM DTE. For the titration measurement, data were recorded with Fluoromax-4 spectrophotometer (HORIBA Jobin Yvon, Munich, Germany) with excitation and emission wavelengths at 530 and 580 nm for TAMRA-labelled INPP5E peptide and at 495 and 520 nm for fluorescein-labelled Rheb peptide. The kinetic measurements were monitored by a stopped-flow apparatus (Applied Photophysics) in the polarization mode using an excitation wavelength of 366 nm and filter with 420 nm cutoff for mantGppNHp bound Arl protein, excitation wavelength of 495 nm and filter with 520 nm cutoff for fluorescein-labelled Rheb peptide and excitation wavelength of 530 nm and filter with 570 nm cutoff for TAMRA-labelled INPP5E peptide. Data analysis was done with GraFit 5.0 program (Erithracus Software). Concentrations used for each experiment are indicated in the corresponding figure legend.

### Cell culture and stable cell line generation

Mouse renal epithelial cells from the inner medullary collecting duct containing a stably integrated FRT cassette (IMCD3 Flp-In, kind gift from M.V. Nachury lab; Flp-In cell line technology by Life technologies) were cultured at 37 °C and 5% CO_2_ in DMEM/F-12, HEPES (Life technologies) complemented with 10% fetal bovine serum and 2 mM L-Glutamine. Stable cell lines were generated as previously described^43^,^44^. Briefly, IMCD3 cells were seeded in six-well plates at a density of 100,000 cells per well. On the following day the cells with a confluence of 40–60% were cotransfected with the pG-LAP3 vector (Addgene) containing the gene of interest and pOG44 vector (Life technologies) encoding the FLP recombinase using Lipofectamine 2,000 (Life technologies). Transfected cells were selected with hygromycine in a concentration of 100–200 μg ml^−1^ complemented culture medium. Expression of the respective proteins was proven by immunoblotting with an anti-GFP antibody (1:500; Santa Cruz Biotechnology sc-9996).

### Immunostaining and microscopy

IMCD3 cells stably expressing GFP-tagged protein were plated on poly-L-lysine coated coverslips in six-well plates, each well containing 100,000 cells. Twenty-four hours later, cilia were induced by 48 h serum starvation. Cells were washed in PBS and fixed with 4% formaldehyde in cytoskeletal buffer (2,75 M NaCl, 100 mM KCl, 25 mM Na_2_HPO_4_, 8 mM KH_2_PO_4_, 40 mM MgCl_2_, 40 mM EGTA, 100 mM PIPES, 100 mM Glucose, pH 6.0) for 20 min. After two washes with PBS cells were permeabilized with 0.3% Triton X100 in cytoskeletal buffer for 10 min. Cells were rinsed in 0.1% Tween20 in PBS and blocked in 10% FBS in PBS for 30 min. For immunostaining of primary cilia, mouse 6-11B-1 anti-acetylated tubulin antibody (1:5,000; Sigma T6793) in 10% FBS in PBS was incubated overnight at 4 °C. Alexa Fluor 647 anti-mouse secondary antibody (1:800; Life technologies A-31571) was added for 45 min at room temperature after washing four times with 0.1% Tween20 in PBS. Coverslips were rinsed three times in 0.1% Tween20 in PBS and afterwards in PBS. Nuclei were stained with DAPI (Serva), diluted 1:10,000 in PBS for 1 min. After three washes with PBS, coverslips were fixed on glass slides with Mowiol (Merck). Images were taken using an Olympus IX81 microscope with a CCD camera and a 60x NA 1.35 oil immersion objective.

### Knockdown experiment

The INPP5E(WT) stable cell line was plated on poly-L-lysine coated coverslips in six-well plates at a density of 100,000 cells per well. After 24 h cells were transiently transfected with Lipofectamine 2,000 with siRNAs directed against mouse *Arl3* and a negative control siRNA, following the manufactureŕ;s recommendations. The siRNAs against *Arl3* and for a negative control were provided from Qiagen with the following sequences: for *Arl3* (sense: 5′-GGGUCAGGAACUAACGGAATT-3′, antisense: 5′-UUCCGUUAGUUCCUGACCCGT-3′); for negative control (sense: 5′-UUCUCCGAACGUGUCACGUdTdT-3′, antisense: 5′-ACGUGACACGUUCGGAGAAdTdT-3′). Eighty-four hours later, cells were serum-starved for 24 h and subsequently treated for immunofluorescence microscopy as described before. Image collection was performed utilizing identical settings for every sample.

### GST pull-down assay

IMCD3 cells stably expressing GFP-INPP5E(WT) or GFP-INPP5E(KS) were lysed in lysis buffer containing 75 mM Hepes pH 7.5, 150 mM KCl, 1.5 mM EGTA, 1.5 mM MgCl_2_, 15% glycerol, 0.2% NP-40 and one protease inhibitor cocktail tablet (Roche). Cell lysates were cleared and supernatants were incubated for 1 h at 4 °C with 100 μl GSH-beads conjugated with 20 μM GST-PDE6δ. For the release assay, 20 μM of either Arl2 or Arl3 were added to the previous mixture and incubated for further 1 h at 4 °C. After 5 times washing with the lysis buffer, the complexes were analysed by western blotting using anti-GFP antibody (1:500; Santa Cruz Biotechnology sc-9996) and anti GST (1:5,000; home source). Full scans of western blots are provided in Supplementary Fig. 6.

## Additional information

**Accession codes**: The X-ray crystallographic coordinates for structures reported in this study have been deposited at the Protein Data Bank (PDB), under the accession code 5F2U.

**How to cite this article:** Fansa, E. K. *et al*. PDE6δ-mediated sorting of INPP5E into the cilium is determined by cargo-carrier affinity. *Nat. Commun.* 7:11366 doi: 10.1038/ncomms11366 (2016).

## Supplementary Material

Supplementary Information Supplementary Figures 1-6 and Supplementary Table 1
