# Supplementary material for: PDE6δ-mediated sorting of INPP5E into the cilium is determined by cargo-carrier affinity
Source: Nat Commun. 2016 Apr 11;7:11366. doi: 10.1038/ncomms11366 (PMC5512577; doi:10.1038/ncomms11366)
Supplement: Supplementary Information — Supplementary Figures 1-6 and Supplementary Table 1 [file ncomms11366-s1.pdf]

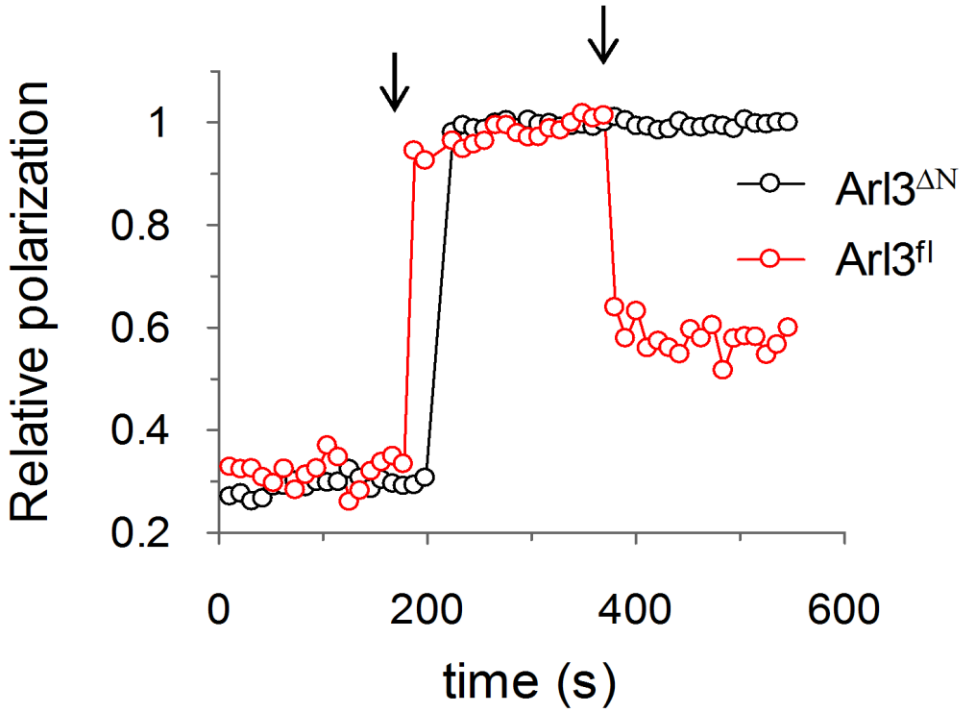

Supplementary Figure 1: INPP5E release is dependent on the N-terminal helix of Arl3•GTP. Fluorescence polarization measurements of 0.2 µM TAMRA-labeled INPP5E peptide followed by addition of 0.2 µM PDE6δ (arrow) and the addition of 5 µM Arl3^{fl} or Arl3$^{\Delta N}$ (arrow).

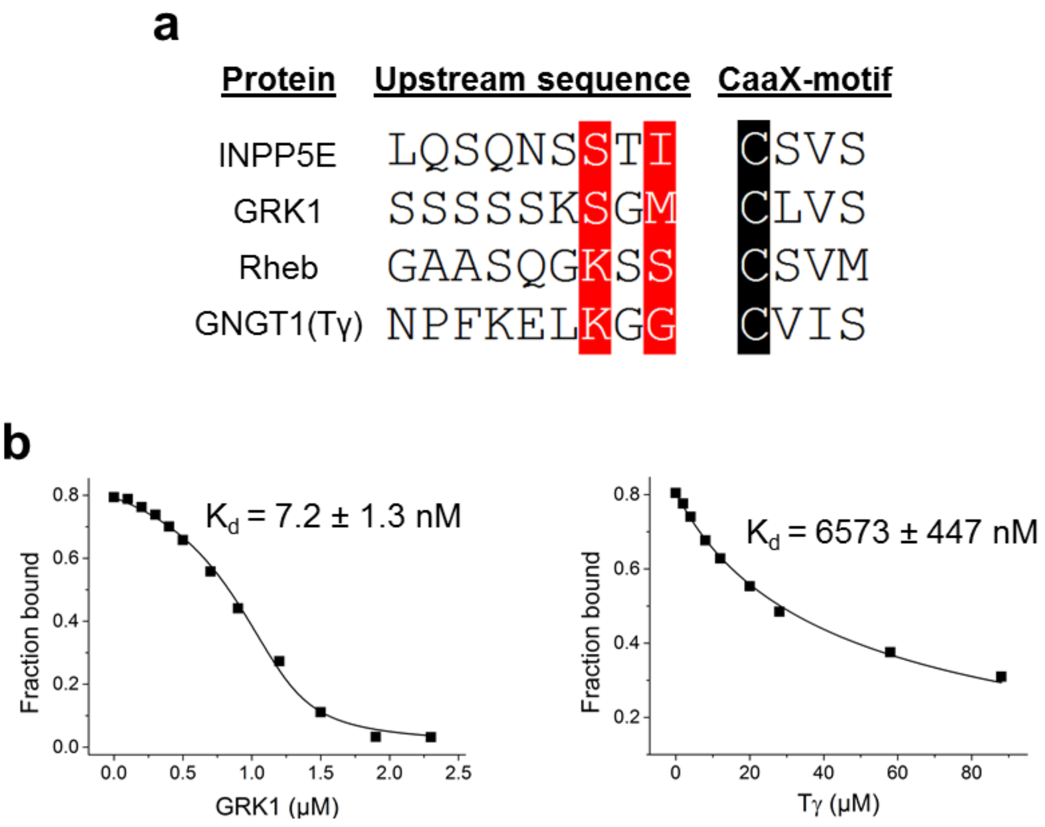

Supplementary Figure 2: Affinity of GRK1 and Tγ farnesylated peptides to PDE6δ. (a) Sequence alignment of C-terminal part of PDE6δ high affinity binding partners (INPP5E and GRK1) and low affinity binding partner (Rheb and Tγ). The prenylated cysteine is highlighted in black; residues at the -1 and -3 positions upstream of the cysteine are highlighted in red **(b)** Titrations of a complex between 0.5 µM FITC-labeled Rheb peptide and 1 µM PDE6δ with increasing concentrations of GRK1 (left) and Tγ (right) peptides. Titration data were fitted with a competition model.

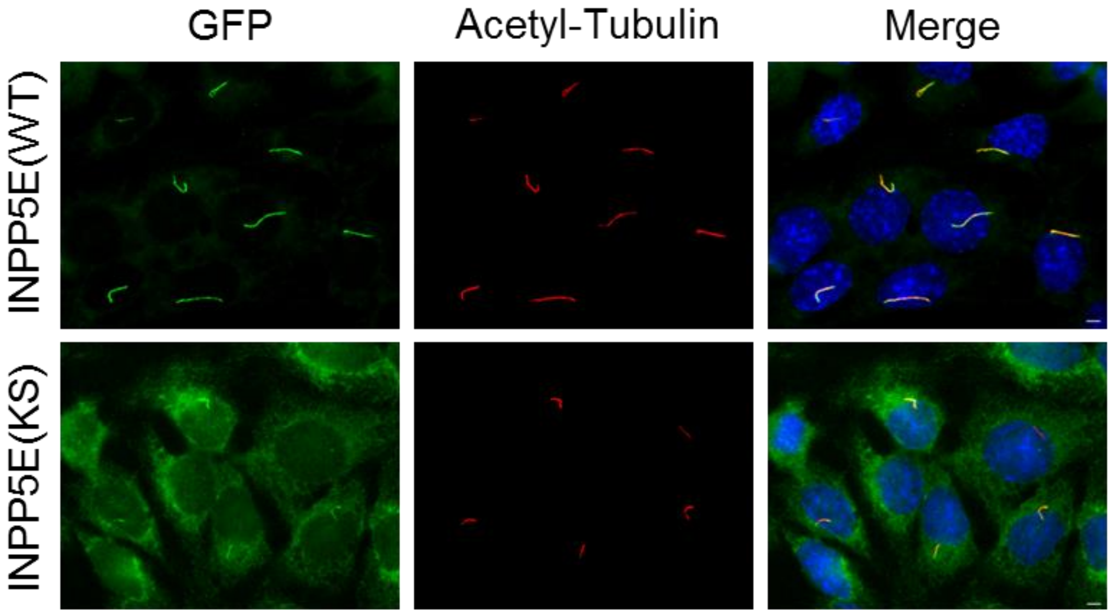

Supplementary Figure 3: Mislocalization of low affinity mutant of INPP5E towards PDE6δ. Localization of either INPP5E(WT) or INPP5E(KS) (green) in IMCD3 cells which were stably transfected with the GFP-tagged proteins. White bar indicates 5 µm.

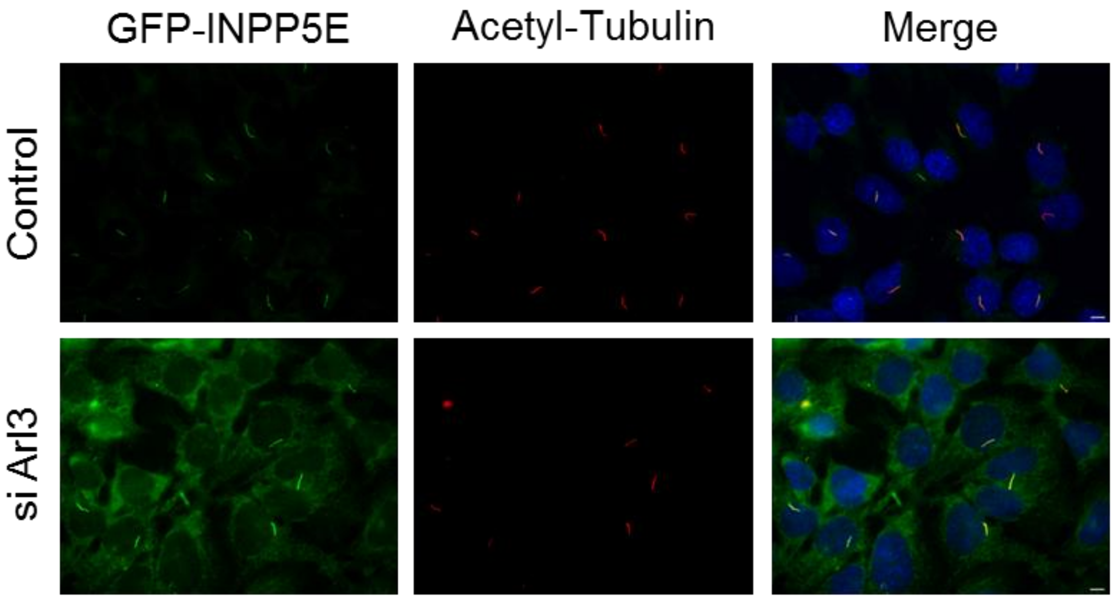

Supplementary Figure 4: Ciliary enrichment of INPP5E is dependent on Arl3. Localization of INPP5E (green) in IMCD3 cells which were stably transfected with the GFP-tagged protein followed by the transfection with either control siRNA or siRNA directed against *Arl3*. White bar indicates 5 µm

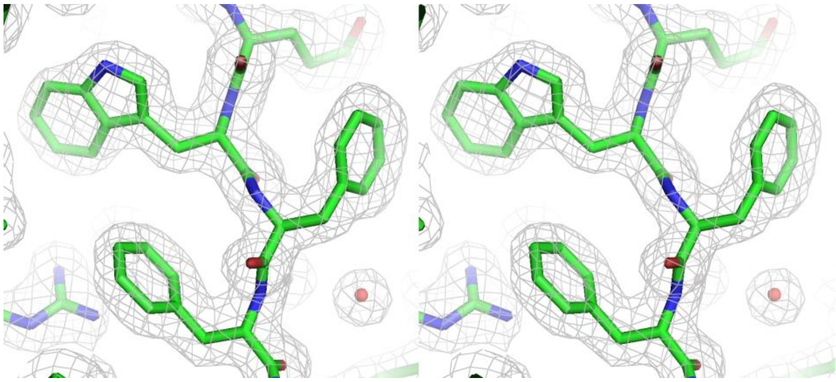

Supplementary Figure 5: A stereo image of a portion of the $2F_o$–F_c electron density map. Representative electron density, as a cross-eyed stereo pair at 1σ level around Try90, Phe91 and Phe92 of F-INPP5E-peptide•PDE6δ complex structure.

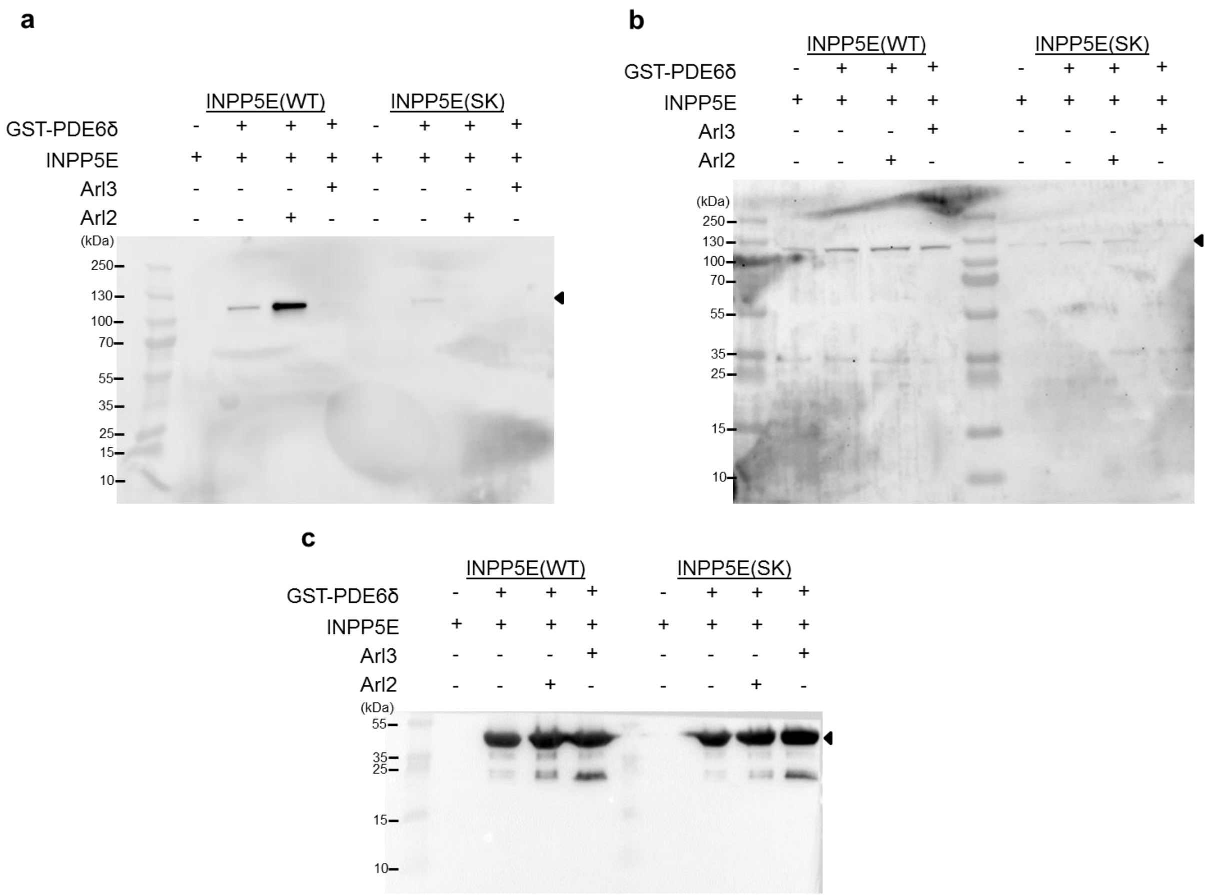

Supplementary Figure 6: Full scans of western blots used in the main figure. (a) Blot against GFP-INPP5E from the GST pull-down. **(b)** Blot against GFP-INPP5E from the total cell lysate. **(c)** Blot against GST-PDE6δ from the GST pull-down. Black arrows indicate the target proteins.

Supplementary table 1: Data collection and refinement statistics (molecular replacement).

	F-INPP5E-peptide•PDE6δ
**Data collection**	
Space group	$C222_1$
Cell dimensions	
a, *b*, *c* (Å)	77.47, 81.20, 117.21
α, β, γ (°)	90.00, 90.00, 90.00
Resolution (Å)	19.53-1.85 (1.9-1.85)
R_{sym} or R_{merge}	9.7 (67.2)
$I / \sigma I$	10.42 (3.09)
Completeness (%)	99.8 (100.0)
Redundancy	6.39 (6.58)
R_{meas}	10.5 (69.1)
R_{pim}	4.1 (26.0)
**Refinement**	
Resolution (Å)	19.53-1.85 (1.9-1.85)
No. reflections	31895 (2396)
No. collected reflections	203615 (15773)
R_{work} / R_{free}	17.3/20.7 (24.0/29.6)
No. atoms	
Protein	2434
Ligand/ion	112
Water	89
B-factors	
Protein	34.0
Ligand/ion	39.66
Water	39.84
R.m.s. deviations	
Bond lengths (Å)	0.0201
Bond angles (°)	2.0109
Ramachandran plotstatistics	
Favoured region (%)	98.3

Allowed region (%)	1.7
Outlier region (%)	0.0
**PDB code**	5F2U

Numbers in parentheses represent the highest-resolution bin.
